# ‘Masked apical sparing’ in wild-type transthyretin cardiac amyloidosis complicated by aortic valve stenosis: a case report

**DOI:** 10.1093/ehjcr/ytaf471

**Published:** 2025-09-20

**Authors:** Kei Matsumoto, Keisuke Matsuo, Takahide Arai, Shintaro Nakano

**Affiliations:** Department of Cardiology, International Medical Center, Saitama Medical University, 1397-1 Yamane, Hidaka-Shi, Saitama 350-1298, Japan; Department of Cardiology, International Medical Center, Saitama Medical University, 1397-1 Yamane, Hidaka-Shi, Saitama 350-1298, Japan; Department of Cardiology, International Medical Center, Saitama Medical University, 1397-1 Yamane, Hidaka-Shi, Saitama 350-1298, Japan; Department of Cardiology, International Medical Center, Saitama Medical University, 1397-1 Yamane, Hidaka-Shi, Saitama 350-1298, Japan

**Keywords:** Case report, Aortic stenosis, Transcatheter aortic valve implantation, Transthyretin cardiac amyloidosis, Global longitudinal strain

## Abstract

**Background:**

Wild-type transthyretin cardiac amyloidosis (ATTRwt-CA) may accompany aortic stenosis (AS) in older adults. The coexistence of ATTRwt-CA and AS is associated with increased mortality and a higher incidence of heart failure hospitalization compared to AS alone. Apical sparing, manifested with a preserved apical longitudinal strain by echocardiography, is a cornerstone of diagnosing ATTRwt-CA; however, the role of this parameter in the diagnosis and follow-up of patients with AS complicated with ATTRwt-CA remain.

**Case summary:**

We report a case of ATTRwt-CA complicated by AS that was successfully treated with transcatheter aortic valve implantation (TAVI) and tafamidis, a tetramer stabilizer for transthyretin. The patient exhibited a favourable clinical course, highlighting the potential benefit of this combined therapeutic approach. Notably, characteristic features suggestive of apical sparing, which were completely absent before TAVI, emerged 10 months after the interventional afterloading.

**Discussion:**

This case indicates the potential benefit of combining tafamidis therapy with TAVI in patients with ATTRwt-CA and AS. Serial assessment of global longitudinal strain (GLS) provides valuable insights into myocardial function beyond the ejection fraction. The absence of the apical sparing sign prior to TAVI and the improvement of apical LS following the interventional afterloading suggest the risk of overlooking the characteristic GLS features of ATTRwt-CA when complicated with AS.

Learning points

- Severe aortic stenosis can mask the apical sparing pattern typical of wild-type transthyretin cardiac amyloidosis.
- Transcatheter aortic valve implantation (TAVI) may unmask apical sparing by relieving afterload-induced strain suppression.
- Combined TAVI and tafamidis therapy offers complementary functional and prognostic benefits.

## Introduction

Wild-type transthyretin cardiac amyloidosis (ATTRwt-CA) is a progressive, infiltrative cardiomyopathy caused by the deposition of misfolded transthyretin-derived amyloid fibrils, predominantly produced in the liver. Wild-type transthyretin cardiac amyloidosis has recently been recognized as an important comorbidity in older patients with aortic stenosis (AS); it occurs in up to 6% of patients aged >65 years undergoing surgical aortic valve replacement^1^ and in ∼16% of patients undergoing transcatheter aortic valve implantation (TAVI), as detected by 99mTc-pyrophosphate scintigraphy.^2^

The coexistence of ATTRwt-CA and AS is associated with increased mortality and a higher incidence of heart failure hospitalization compared to AS alone.^3^ However, the pathophysiological relationship between AS and ATTRwt-CA remains incompletely understood. Amyloid infiltration of the aortic valve and myocardium may contribute to progressive valvular and myocardial dysfunction. Transcatheter aortic valve implantation improves survival in patients with AS, with 5- and 10-year survival rates of 67%–68% and 27%, respectively; however, in patients with coexisting ATTRwt-CA, the optimal therapeutic approach remains to be defined.^4^ Tafamidis, a transthyretin stabilizer, has demonstrated significant reductions in all-cause mortality and cardiovascular-related hospitalizations in patients with transthyretin amyloid cardiomyopathy, as shown in the ATTR-ACT trial.^5^ However, the role of tafamidis in patients with dual pathology involving AS remains unclear.

Global longitudinal strain (GLS), assessed via speckle-tracking echocardiography, is a sensitive marker of early myocardial dysfunction. The characteristic apical sparing pattern, a preserved longitudinal strain in the apical segments relative to the basal and midventricular segments, is a hallmark of cardiac amyloidosis, particularly ATTRwt-CA.^6^ Nonetheless, the reliability of this finding in the setting of pressure-overloaded myocardium due to AS is not well established. In this context, we present a case of advanced AS and ATTRwt-CA treated with both TAVI and tafamidis, in which GLS evaluation revealed that pressure overload masked the classic apical sparing pattern prior to TAVI, which emerged after relief of afterload.

## Summary figure

**Figure:**
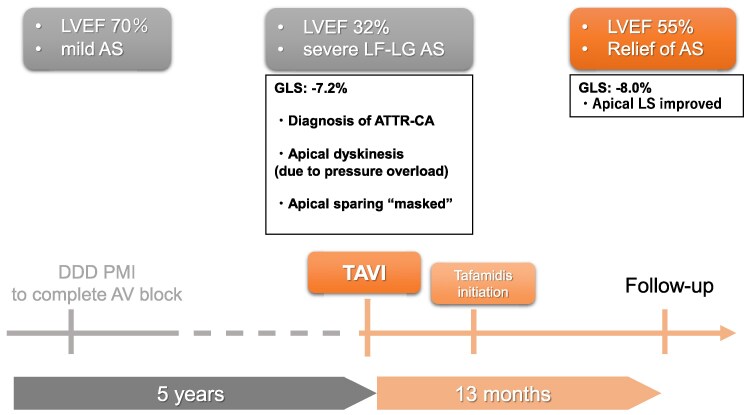
Timeline of clinical events.

## Case presentation

A 78-year-old man with a history of transient atrioventricular block underwent dual-chamber pacemaker implantation. At that time, transthoracic echocardiography (TTE) revealed mild AS with preserved left ventricular ejection fraction (LVEF) of 70%.

Five years later, he presented with dyspnoea on exertion during routine follow-up. Laboratory evaluation showed elevated brain natriuretic peptide levels (979 pg/mL). On physical examination, the patient had a harsh systolic murmur best heard at the right upper sternal border, consistent with severe AS. No peripheral oedema or jugular venous distension was observed. The ECG findings showed no significant changes compared to those after pacemaker implantation. Transthoracic echocardiography revealed LVEF decline to 32% and severe AS with a calculated aortic valve area of 0.77 cm². The patient was referred to our centre for further evaluation of low-flow, low-gradient (LF-LG) AS and possible pacing-induced cardiomyopathy. Serum immunofixation and free light chain analysis indicated a κ (kappa) to λ (lambda) ratio of 2.65, consistent with a polyclonal pattern. Genetic testing for transthyretin (TTR) mutations was performed to rule out hereditary transthyretin amyloidosis (ATTRv).

**Figure 1:**
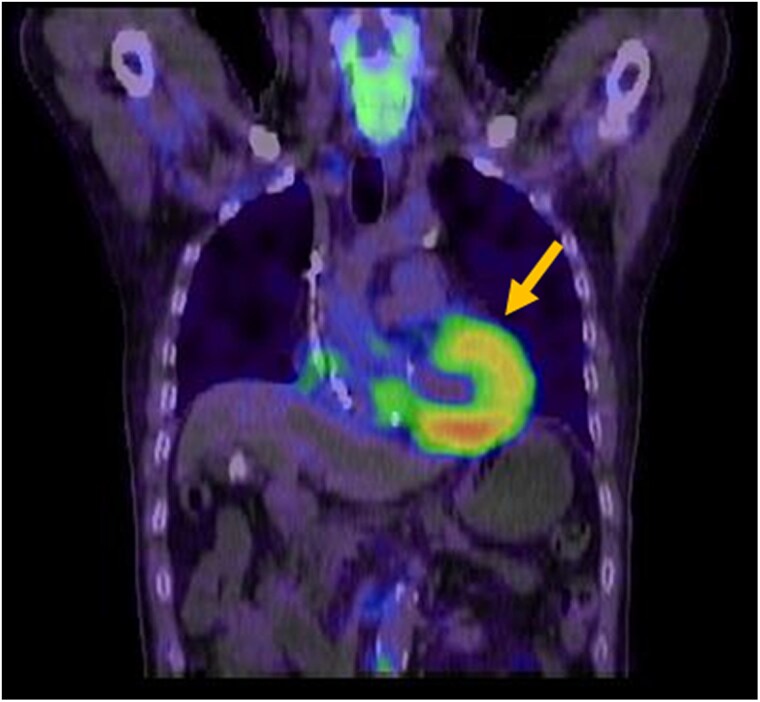
99mTc-pyrophosphate scintigraphy findings: grade 3 uptake is observed (arrow).

**Figure 2:**
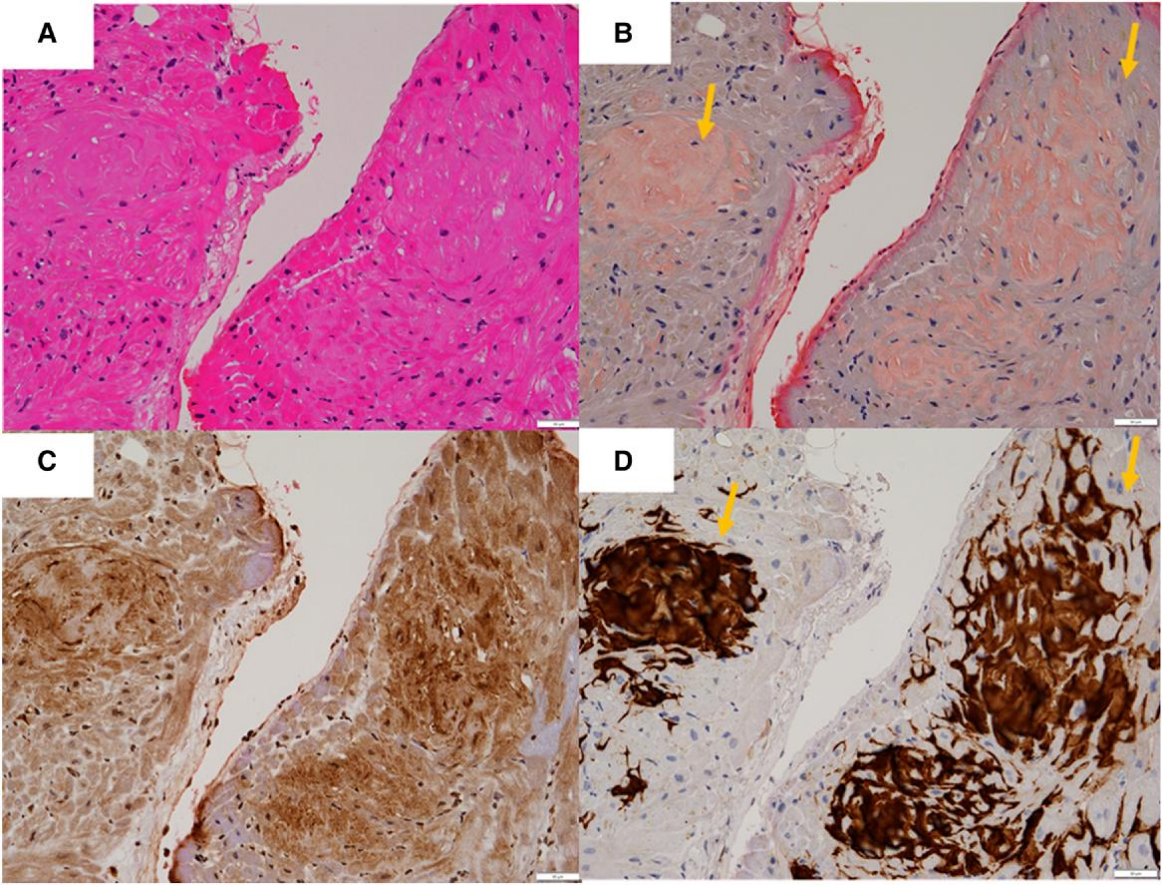
The endomyocardial biopsy findings. (*A*) Haematoxylin and eosin-stained sections of the endocardium indicated deposition of a pale eosinophilic amorphous material. (*B*) The deposition of pale eosinophilic amorphous material was stained with a Direct Fast Scarlet and was positive for amyloid A prealbumin (*C*, *D*).

Cardiac magnetic resonance imaging demonstrated concentric left ventricular hypertrophy, with an interventricular septal thickness of 14 mm and posterior wall thickness of 12 mm. 99mTc-pyrophosphate scintigraphy revealed grade 3 myocardial uptake, strongly suggestive of ATTRwt-CA (*Figure 1*). Endomyocardial biopsy from the right ventricle confirmed amyloid deposition with positive direct fast scarlet staining, and laboratory testing revealed positive prealbumin and weakly positive amyloid A, supporting a diagnosis of transthyretin cardiac amyloidosis (*Figure 2*).

Based on heart failure hospitalization history and interventricular septal thickness > 12 mm, the patient met the criteria for tafamidis therapy and TAVI. Transcatheter aortic valve implantation was successfully performed via the right subclavian artery approach under general anaesthesia. The patient completed rehabilitation and was discharged in stable condition. The ECG findings remained unchanged before and after TAVI. Tafamidis 80 mg daily was initiated 2 months post-discharge.

**Figure 3:**
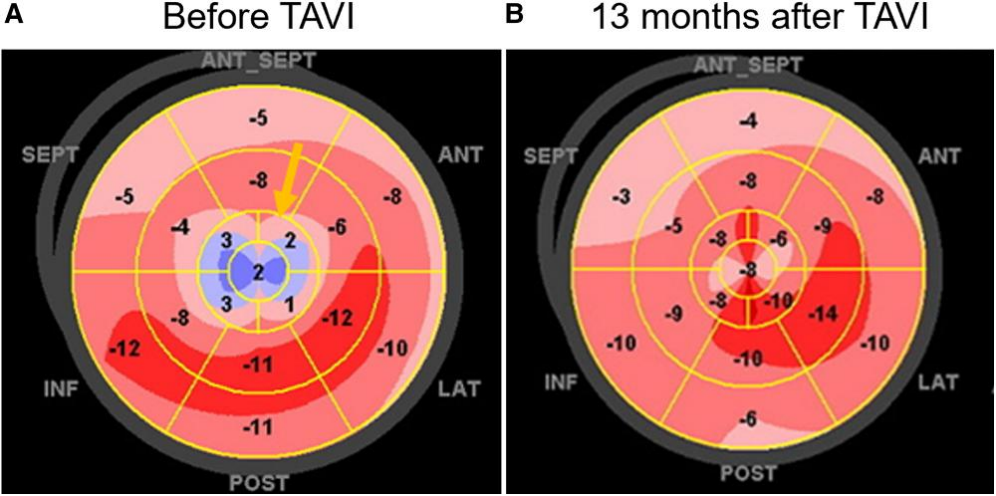
Global longitudinal strain as calculated by transthoracic echocardiogram demonstrating: (*A*) before TAVI and tafamidis administration. Especially, a significant wall motion abnormality was noted in the apical region (arrow). (*B*) Thirteen months after TAVI and tafamidis administration. Wall motion improved at the apex, and apical sparing was not observed.

At 10 and 13 months post-treatment, follow-up TTE revealed improvement in LVEF from 49% to 55%. Global longitudinal strain, initially severely impaired at −7.2%, showed modest improvement to −8.0%. Notably, apical longitudinal strain improved post-TAVI; however, the typical apical sparing pattern was not observed initially but emerged later in follow-up strain imaging (*Figure 3*).

## Discussion

We report a case of ATTRwt-CA complicated by AS, which was managed successfully through a combination of TAVI and tafamidis therapy. This case highlights the complex interplay between amyloid infiltration and pressure overload and how these factors may confound diagnostic imaging markers such as GLS.

Speckle-tracking echocardiography and GLS have emerged as valuable tools in the diagnosis and monitoring of ATTRwt-CA. Among these, the apical sparing pattern, characterized by preserved longitudinal strain in apical segments relative to basal and midventricular segments, is a hallmark of cardiac amyloidosis. This feature is quantitatively expressed as the ratio [average apical LS/(average basal LS + mid LS)], with a cut-off value of >1.0 yielding a sensitivity of 93% and specificity of 82% for cardiac amyloidosis.^6^ In our case, however, this typical pattern was not observed prior to TAVI, despite histologically confirmed ATTRwt-CA.

In our case, however, this characteristic pattern was not evident on pre-TAVI imaging, despite histological confirmation of ATTRwt-CA. This absence can likely be attributed to pressure overload from severe AS. A large retrospective study in patients with severe AS undergoing TAVI (*n* = 620) demonstrated improvement in GLS, including apical segments, at 6-month follow-up.^7^ These findings suggest that chronic left ventricular afterload results in diffuse myocardial strain impairment, which can mask regional differences and diminish the diagnostic sensitivity of strain-based markers. In our patient, GLS before TAVI showed markedly reduced apical strain, likely reflecting + apical dyskinesis from pressure overload. Following TAVI, regional apical function improved, and the apical sparing pattern became evident, supporting the hypothesis that AS-induced afterload can obscure classic imaging findings of ATTRwt-CA.

It is important to note, however, that the apical sparing pattern is not entirely specific to ATTR-CA. A prospective study of patients with severe AS (*n* = 157) without evidence of amyloidosis demonstrated that 15% exhibited this pattern, indicating that it may also be present in the context of pressure overload alone.^8^

Additionally, our patient had undergone transvenous permanent pacemaker implantation (TV-PMI), with the right ventricular lead placed at the apex. Previous data indicate that RV apical pacing is associated with greater reductions in both GLS and regional apical strain compared to RV septal pacing over a 6-month period.^9^ Thus, right ventricular apical pacing may act as a modifying factor influencing apical strain patterns.

Tafamidis, a transthyretin tetramer stabilizer, has been shown to reduce all-cause mortality and cardiovascular-related hospitalizations in patients with transthyretin amyloid cardiomyopathy, as demonstrated in the ATTR-ACT trial.^5^ However, prior reports suggest that tafamidis has a limited effect on improving GLS parameters. Studies have shown that while tafamidis effectively halts disease progression, it does not significantly reverse existing myocardial deformation, and a 1-year follow-up generally reveals no significant change in global strain.^5^

In contrast, unloading the ventricle via TAVI can improve both systolic and diastolic myocardial function. Improvements in GLS following TAVI have been reported in patients with AS, and these changes are often more pronounced than those seen with pharmacologic therapy alone.^10^ In our case, the modest improvement in overall GLS (from −7.2% to −8.0%) and the marked improvement in apical segment strain suggest that TAVI was the primary driver of functional recovery. This result supports the hypothesis that AS-related afterload not only contributes to cardiac dysfunction but may also mask the regional strain features critical for diagnosing ATTRwt-CA.

Early abnormalities in GLS can serve as sensitive markers of myocardial dysfunction, often preceding reductions in LVEF. Serial GLS monitoring enables the earlier detection of functional changes and facilitates longitudinal surveillance of treatment responses.^11^ Therefore, integrating GLS into the diagnostic and follow-up algorithms is particularly useful in cases of overlapping cardiac pathologies, such as ATTRwt-CA.

In conclusion, this case illustrates that pressure overload from AS can obscure the hallmark apical sparing pattern of ATTRwt-CA on GLS imaging. The relief of afterload through TAVI may unmask these features, thereby enhancing the diagnostic accuracy. While tafamidis contributes to long-term disease stabilization, its short-term impact on strain parameters appears limited. The combination of TAVI and tafamidis may offer complementary therapeutic benefits, particularly in complex cases in which diagnostic features are initially masked.

Furthermore, the reported patterns of apical movement and kinetic due to severe AS vary substantially, making them inconclusive. We assume that the apical movement behaves on case-by-case basis, dependent on the severity, stage, diastolic and systolic function, or combined of these multifactorially. In our case, the pre-TAVI apical LS was markedly reduced and accompanied by dyskinetic motion. We acknowledge the limitation of a single-case based finding, it may be reasonable that this characteristic feature was contributed by pressure overload, as the finding apparently disappeared after the TAVI, literally interventional afterload reduction.

## Patient’s perspective

The patient expressed satisfaction with the clinical improvement following TAVI and initiation of tafamidis, particularly noting an improvement in exercise tolerance and quality of life.

## Data Availability

The data underlying this article are not publicly available due to patient privacy concerns but are available from the corresponding author upon reasonable request.

## Lead author biography

Kei Matsumoto 2019, M.D., Faculty of Medicine, Oita University. 2019.4—Resident in the Department of Internal Medicine, Saitama Medical University Hospital, Saitama, Japan. 2021.4—Assistant Professor in the Division of Cardiology, The Department of Internal Medicine, Saitama Medical University International Medical Center, Saitama, Japan. 2023.4—Resident in the Division of Cardiology, Saitama Red Cross Hospital, Saitama, Japan. 2024.4—Assistant Professor in the Division of Cardiology, The Department of Internal Medicine, Saitama Medical University International Medical Center, Saitama, Japan.
